# Antipsychotics and the risk of diabetes and death among adults with serious mental illnesses

**DOI:** 10.1017/S0033291723001502

**Published:** 2023-12

**Authors:** Jason Poulos, Sharon-Lise T. Normand, Katya Zelevinsky, John W. Newcomer, Denis Agniel, Haley K. Abing, Marcela Horvitz-Lennon

**Affiliations:** 1 Department of Health Care Policy, Harvard Medical School, Boston, MA, USA; 2 Department of Biostatistics, Harvard TH Chan School of Public Health, Boston, MA, USA; 3 Thriving Mind South Florida, Miami, FL, USA; 4 Department of Psychiatry, Washington University School of Medicine, St. Louis, MO, USA; 5 RAND Corporation, Santa Monica, CA, USA; 6 RAND Corporation, Boston, MA, USA; 7 Department of Psychiatry, Cambridge Health Alliance and Harvard Medical School, Cambridge, MA, USA

**Keywords:** Serious mental illness, antipsychotic, type 2 diabetes, mortality, robust causal estimation

## Abstract

**Background:**

Individuals with schizophrenia exposed to second-generation antipsychotics (SGA) have an increased risk for diabetes, with aripiprazole purportedly a safer drug. Less is known about the drugs' mortality risk or whether serious mental illness (SMI) diagnosis or race/ethnicity modify these effects.

**Methods:**

Authors created a retrospective cohort of non-elderly adults with SMI initiating monotherapy with an SGA (olanzapine, quetiapine, risperidone, and ziprasidone, aripiprazole) or haloperidol during 2008–2013. Three-year diabetes incidence or all-cause death risk differences were estimated between each drug and aripiprazole, the comparator, as well as effects within SMI diagnosis and race/ethnicity. Sensitivity analyses evaluated potential confounding by indication.

**Results:**

38 762 adults, 65% White and 55% with schizophrenia, initiated monotherapy, with haloperidol least (6%) and quetiapine most (26·5%) frequent. Three-year mortality was 5% and diabetes incidence 9.3%. Compared with aripiprazole, haloperidol and olanzapine reduced diabetes risk by 1.9 (95% CI 1.2–2.6) percentage points, or a 18.6 percentage point reduction relative to aripiprazole users' unadjusted risk (10.2%), with risperidone having a smaller advantage. Relative to aripiprazole users' unadjusted risk (3.4%), all antipsychotics increased mortality risk by 1.1–2.2 percentage points, representing 32.4–64.7 percentage point increases. Findings within diagnosis and race/ethnicity were generally consistent with overall findings. Only quetiapine's higher mortality risk held in sensitivity analyses.

**Conclusions:**

Haloperidol's, olanzapine's, and risperidone's lower diabetes risks relative to aripiprazole were not robust in sensitivity analyses but quetiapine's higher mortality risk proved robust. Findings expand the evidence on antipsychotics' risks, suggesting a need for caution in the use of quetiapine among individuals with SMI.

## Introduction

In the U.S., second-generation antipsychotics (SGAs) are the de-facto first line antipsychotic agents for the treatment of schizophrenia, displacing first-generation antipsychotics (FGAs) for this indication. SGAs are also widely used in the treatment of major affective disorders, with selected drugs having U.S. Food and Drug Administration (FDA) approval for the treatment of bipolar I disorder and treatment-resistant major depressive disorder (MDD).

Compared to the general population, people with serious mental illnesses (SMI; schizophrenia, bipolar I disorder, severe MDD) have a higher risk for type 2 diabetes, hereafter diabetes, cardiovascular disease, and other cardiometabolic morbidity (Lindekilde et al., 2022; Momen et al., 2020; Takayanagi et al., 2021; Wimberley et al., 2022). Evidence exists that for diabetes and other insulinopathies, this excess risk may stem at least in part from shared genetic loading as reflected in familial aggregation (Huang et al., 2019; Su et al., 2022; van Welie et al., 2013), with dysregulation of insulin signaling as a potential mechanism for both sets of disorders (Fanelli et al., 2022). Studies have found that antipsychotics, particularly SGAs, contribute to this risk relative to FGAs or to no drug. The preponderance of evidence, much of it derived from populations with schizophrenia, suggests that olanzapine and clozapine confer a high risk for cardiometabolic morbidity and its risk factors (weight gain and metabolic dysregulation), quetiapine and risperidone confer an intermediate risk, and aripiprazole confers a lower risk (Holt & Mitchell, 2015; Mazereel, Detraux, Vancampfort, van Winkel, & De Hert, 2020). In the U.S., concern about the association between SGAs and diabetes grew through the early 2000′s, culminating in an FDA warning about the increased risk of diabetes associated with all SGAs and in professional societies raising alarms about this risk (American Diabetes Association et al., 2004). The significance of diabetes among people with SMI stems from its higher prevalence and severity relative to those without SMI (Holt & Mitchell, 2015) and its contribution to their risk of cardiovascular disease (Osborn et al., 2008) and premature mortality (Walker, McGee, & Druss, 2015).

Prior studies on the association between SGAs and diabetes risk among individuals with SMI have some limitations, however. Randomized trials have been mainly designed to assess antipsychotic drugs' efficacy (Holt & Mitchell, 2015) and safety assessments have focused on risk factors for diabetes rather than diabetes itself (Pillinger et al., 2020). Otherwise, most evidence arises from observational studies designed using a variety of comparison arms: any SGA (Smith et al., 2008) or specific SGAs (Citrome et al., 2007) *v.* any FGA; or antipsychotic drugs *v.* specific FGAs, typically haloperidol (Hartling et al., 2012), or *v.* no antipsychotic drug (Kessing, Thomsen, Mogensen, & Andersen, 2010). Because cardiometabolic morbidity risk may vary by specific SGAs, results from pooled analyses may mask effects. Studies that compare SGA outcomes to individuals receiving no antipsychotic drug treatment inform about whether to prescribe rather than which drug to prescribe; while the former is perhaps appropriate for individuals with affective disorders, it is almost never appropriate for individuals with schizophrenia.

SGAs and haloperidol have been associated with a higher mortality risk among elderly adults with dementia. This evidence, produced both by randomized trials and observational studies, suggests that the risk is highest for haloperidol (Reus et al., 2017). Evidence on antipsychotic drug mortality risk specific to individuals with SMI comes largely from observational studies, most focused on schizophrenia, with few having adequate comparators. While two studies compared SGAs or FGAs to no antipsychotic (Taipale et al., 2020; Tiihonen & Taipale, 2019), a third compared antipsychotic monotherapy to antipsychotic polypharmacy (Katona, Czobor, & Bitter, 2014). We are aware of only one recently published study that conducted pairwise comparisons between multiple antipsychotic drugs and oral paliperidone (Tang et al., 2022), and of one study among adults with treatment-resistant MDD that compared augmentation with a SGA to a second antidepressant (Gerhard et al., 2020). Across these studies conducted in SMI cohorts, aripiprazole appeared among the safest, with haloperidol's risk comparatively higher.

In this paper, we used novel causal inference methods in a large, racially/ethnic diverse population to re-examine the effect of commonly used antipsychotic drugs on the risk of diabetes and mortality in a publicly insured U.S. adult population with SMI. Because of aripiprazole's purportedly lower risks, we compared each antipsychotic drug to aripiprazole. We estimated antipsychotic drug effect heterogeneity associated with race/ethnicity and primary diagnosis because little is known on whether these characteristics modify the drugs' safety effects.

## Methods

### Data sources, study cohort, and design

In the U.S., Medicaid is a joint federal and state program that provides health coverage to low-income adults and disabled individuals, while Medicare is a federal health insurance program for those 65 years or older and disabled non-elderly adults with past employment. Some individuals may be dually enrolled in Medicaid and Medicare due to a qualifying disability (hereafter dual eligibles). We used administrative health care billing data from both programs to assemble information on eligibility, demographic characteristics, diagnoses, and service and pharmacy utilization; for dual eligibles, information from both Medicare and Medicaid files was linked.

The study was reviewed and approved by the authors' IRBs. We followed the STROBE reporting guideline (see online Supplementary Table S3).

We included adults aged 18–64 of non-Latinx Black (hereafter Black), Latinx, non-Latinx White (hereafter White), or other race/ethnicity having dual Medicaid-Medicare and Medicare-only coverage, residing in any of seven states (California, Georgia, Iowa, Mississippi, Oklahoma, South Dakota, and West Virginia), who (a) between 1 July 2008 and 30 June 2013, filled at least one prescription for any of the most commonly utilized SGAs (quetiapine, risperidone, aripiprazole, olanzapine, ziprasidone) or haloperidol, and (b) were diagnosed with an SMI, i.e., schizophrenia, bipolar I disorder, or severe MDD (as a proxy for treatment-resistant MDD). These states were selected for their racial/ethnic diversity.

We identified individuals who met criteria for antipsychotic drug monotherapy with any of the six (‘index’) drugs and who were *relatively new* antipsychotic drug users. Monotherapy was defined as ⩾ two fills for the same index antipsychotic drug totaling a minimum supply of 31 days during a 90-day period, with the date of the first fill denoted the index date. We defined *relatively new* antipsychotic drug users as individuals who in the 6-month period preceding the index date (hereafter, the pre-period) had no fills for any antipsychotic drug other than their index drug.

The final cohort included (1) individuals with at least 6 months of continuous enrollment in the pre-period (used to assess for confounders) and at least 6 months of continuous enrollment in the period immediately following the index date (post-period). We included those who died during the post-period if they were continuously enrolled up to the month prior to the month of death; and (2) those not having diabetes conditions other than type 1 diabetes, other cardiometabolic morbidity (dyslipidemia, hypertension, and cardiovascular disorders) associated with diabetes risk or death, or conditions such as polycystic ovaries syndrome whose management frequently involves use of antidiabetic drugs during the pre-period. Individuals were followed from the index date up to 3 years regardless of the duration of the monotherapy, unless a censoring event was observed, which in hierarchical order, included (a) end of the study period (31 December 2013), (b) turning 65 years, or (c) loss of insurance coverage. Individuals could only contribute one episode to the cohort (see online Supplementary material, A.1 and A.2).

### Measures

#### Outcomes

The primary outcomes were newly diagnosed type 2 diabetes or all-cause death. Type 2 diabetes was ascertained with ICD-9 diagnosis codes observed as (a) a primary diagnosis in ⩾ one inpatient discharge claims, or (b) primary or secondary diagnosis in ⩾ two outpatient claims during a 12-month period, or in one outpatient claim if a National Drug Code (NDC) for an oral antidiabetic drug. All-cause death was defined based on variables denoting the person's date of death available in the datasets.

#### Treatment

Our treatment variables were indicators for quetiapine, risperidone, aripiprazole, olanzapine, ziprasidone, or haloperidol monotherapy.

#### Subgroups

We categorized patients by their primary SMI diagnosis (schizophrenia, bipolar I disorder, severe MDD) as well as by race/ethnicity (Black, Latinx, White, or other race/ethnicity which also includes individuals with missing race/ethnicity information).

#### Confounders

Confounders, all measured during the pre-period, included: age^1^^†^ and sex; health status, assesse with three variables: (a) other chronic medical conditions potentially associated with diabetes or having the potential to affect service utilization and thus likelihood of diagnosis (e.g. HIV & other chronic infections, Type 1 diabetes & other endocrine disorders, malignancies), (b) risk factors for cardiometabolic morbidity (e.g. obesity, pre-diabetes), and (c) psychiatric comorbidity (e.g. other affective disorders, post-traumatic stress disorder); service utilization, assessed with nine variables capturing counts of psychiatric, injury-related, and non-psychiatric inpatient days, emergency department visits, and outpatient visits; metabolic testing, which captured lipid or glucose laboratory tests; exposure to drugs with cardiometabolic effects, including antidiabetic drugs, anti-hypertensive drugs, and other drugs with potential weight-related and cardiometabolic effects; exposure to the index antipsychotic drug; payer, based on dual Medicaid-Medicare *v.* Medicare coverage of the index fill; and year of the index fill (see online Supplementary material, A.3).

### Statistical analysis

We estimated the difference in the rate of the primary outcomes between each antipsychotic drug and aripiprazole using targeted minimum loss-based estimation (TMLE), a doubly-robust estimator implemented with machine learning (Schuler & Rose, 2017; van der Laan & Rose, 2011) to reduce the risk of model misspecification (Pirracchio, Petersen, & van Der Laan, 2015). We modified the algorithm to use minimum loss-based estimation with a categorical treatment variable (Poulos et al., 2022). TMLE uses machine learning models trained on potentially risk-modifying patient characteristics to estimate the expected outcome as if all subjects were treated with each antipsychotic drug, by reweighting an initial outcome estimate with a function of estimated treatment probabilities. Average absolute outcome differences were obtained by differencing the TMLE estimates of the expected outcome under each drug and the expected outcome under the comparator (aripiprazole), and average relative outcome differences were obtained by scaling the absolute difference by the observed (unadjusted) outcome rate in the aripiprazole group. 95% confidence intervals (CIs) for the average absolute differences were constructed using the efficient influence curve (Poulos et al., 2022). Data were analyzed using the super learner (Polley, Rose, & van der Laan, 2011; van der Laan, Polley, & Hubbard, 2007), an ensemble method that employs cross-validation to select the optimal weighted average of estimators obtained from a pre-selected library of classification algorithms (see online Supplementary material, A.4). To assess consistency of the main findings, we averaged the estimated differences in the expected outcome rates within the subgroups defined by primary SMI diagnosis and again by race/ethnicity. We made no adjustment for multiplicity of the pairwise comparisons.

### Sensitivity to unmeasured confounding

We identified a subgroup of young (less than 45 years old) individuals having no exposure to the index antipsychotic drug in the pre-period. These individuals are (a) less likely than older individuals or those with prior exposure to the index drug to have developed undetected or uncoded diabetes or its risk factors; and (b) because of no pre-period exposure to the index drug, less information is available to prescribers on early risk signals, suggesting this subgroup is less vulnerable to unmeasured confounding. We averaged the estimates obtained from the full cohort within the subgroup, computing the diabetes incidence and mortality risk differences between each antipsychotic drug and aripiprazole.

## Results

### Characteristics of the study cohort

Our final cohort included 38 762 individuals who were receiving one of the study drugs and were followed for up to 3 years between 2008 and 2013 (or died before the 3-year follow-up) (see online Supplementary Fig. S1). Median follow-up was identical across all drug groups (1094 days), and mean follow-up was nearly identical (998–999 days). The cohort was predominantly White (65%), male (52%), and older than 45 (53%); most were residents of California (55%) and individuals with dual Medicaid-Medicare coverage (74%). The primary SMI diagnosis for most of the cohort was schizophrenia (55%); a sizable minority had comorbidity, with both psychiatric (17.5%) and other chronic medical conditions (21.8%) common. Most individuals (81%) had some pre-period exposure to the index drug, and on average, they were on antipsychotic drugs for 109 days (s.d. = 69.7). Metabolic monitoring with lipid or glucose lab tests was infrequent (18%) (Table 1).

**Table 1.** Cohort characteristics

	Aripiprazole		Haloperidol		Olanzapine		Quetiapine		Risperidone		Ziprasidone		All	
Variable	*N*	%	*N*	%	*N*	%	*N*	%	*N*	%	*N*	%	*N*	%
Individuals	6686	17.2	2328	6.0	6301	16.2	**10 309**	26.5	9897	25.5	3241	8.3	38762	100⋅0
Age category (index date)														
18–30	774	11.6	221	9.5	612	9.7	955	9.3	1213	12.3	396	12.2	4171	10.8
31–45	2530	37.8	722	31.0	2177	34.5	3567	34.6	3413	34.5	1233	38.0	13 642	35.2
>45	3222	48.2	1340	57.6	3385	53.7	5627	54.6	5073	51.3	1565	48.3	20 212	52.1
Female (index date)	3889	58.2	784	33.7	2160	34.3	5756	55.8	3954	40.0	1917	59.1	18 460	47.6
Race/ethnicity (index date)														
Black,	803	12.0	768	33.0	1062	16.9	1370	13.3	2132	21.5	514	15.9	6649	17.1
Latinx	749	11.2	268	11.5	695	11.0	1048	10.2	1102	11.1	325	10.0	4187	10.8
White	4686	70.1	1146	49.2	4030	64.0	7328	71.1	5935	60.0	2224	68.6	25 349	65.4
Other/Missing	448	6.7	146	6.3	514	8.2	563	5.5	728	7.4	178	5.5	2577	6.7
Primary serious mental illness diagnosis														
Schizophrenia	2862	42.8	2026	87.0	4396	69.8	3603	35.0	6799	68.7	1615	49.8	21 301	55.0
Bipolar I disorder	2042	30.5	178	7.7	1167	18.5	3642	35.3	1639	16.6	1053	32.5	9721	25.1
Severe Major depressive disorder	1782	26.6	124	5.3	738	11.7	3064	29.7	1459	14.7	573	17.7	7740	20.0
Health status														
Other chronic medical conditions	1548	23.1	324	13.9	1168	18.5	2712	26.3	1934	19.5	775	23.9	8461	21.8
Risk factors for cardiometabolic morbidity	178	2.7	27	1.2	77	1.2	195	1.9	166	1.7	75	2.3	718	1.9
Psychiatric comorbidity	1250	18.7	271	11.6	886	14.1	2255	21.9	1525	15.4	587	18.1	6774	17.5
Metabolic testing														
Lipid or glucose tests	1246	18.6	300	12.9	989	15.7	2247	21.8	1681	17.0	634	19.6	7097	18.3
Drugs with cardiometabolic effects, any exposure														
Antidiabetic drugs	364	5.4	134	5.8	185	2.9	527	5.1	543	5.5	200	6.2	1953	5.0
Other drugs for cardiometabolic disorders	1798	26.9	516	22.2	1435	22.8	2866	27.8	2295	23.2	904	27.9	9814	25.3
Other drugs with cardiometabolic effects	4775	71.4	1073	46.1	3611	57.3	7508	72.8	5812	58.7	2338	72.1	25 117	64.8
Index antipsychotic drug exposure														
Any exposure	5011	(75.0)	1947	(83.6)	5464	(86.7)	8058	(78.2)	8192	(82.8)	2699	83.3	31 371	80.9
Payer (index date)	***N***	**%**												
Dual	5035	75.3	1629	70.0	4893	77.7	7365	71.4	7399	74.8	2285	70.5	28 606	73.8
Medicare	1651	24.7	699	30.0	1408	22.4	2944	28.6	2498	25.2	956	29.5	10 156	26.2
Index year (index date)														
2008	3110	46.5	1179	50.6	3634	57.7	4937	47.9	5232	52.9	1628	50.2	19 720	50.9
2009	2038	30.5	646	27.8	1501	23.8	2939	28.5	2528	25.5	895	27.6	10 547	27.2
2010^a^	1538	23.0	503	21.6	1166	18.5	2433	23.6	2137	21.6	718	22.1	8495	21.9
	Mean	s.d.	Mean	s.d.	Mean	s.d.	Mean	s.d.	Mean	s.d.	Mean	s.d.	Mean	s.d.
Index antipsychotic drug exposure														
Days	96.0	(71.2)	105⋅4	(68.0)	124⋅6	(65.2)	102⋅2	(70.8)	114⋅7	(68.7)	115⋅7	(68.2)	109⋅3	(69.7)
Service utilization, Mean (s.d.)														
Psychiatric inpatient days	1.4	(7.8)	2.7	(9.6)	2.0	(8.9)	1.9	(7.8)	2.3	(9.4)	1.7	(8.1)	2.0	(8.6)
Injury-related inpatient days	0.0	(0.7)	0.0	(0.5)	0.1	(1.3)	0.1	(1.1)	0.1	(1.6)	0.1	(1.1)	0.1	(1.2)
Non-psychiatric inpatient days	0.5	(3.1)	0.5	(4.7)	0.5	(3.7)	0.9	(5.3)	0.5	(4.1)	0.5	(3.6)	0.6	(4.3)
Psychiatric emergency department visits	0.1	(0.5)	0.2	(0.6)	0.1	(0.6)	0.2	(0.6)	0.1	(0.7)	0.1	(0.5)	0.1	(0.6)
Injury-related emergency department visits	0.1	(0.3)	0.0	(0.3)	0.0	(0.3)	0.1	(0.4)	0.0	(0.2)	0.1	(0.3)	0.1	(0.3)
Non-psychiatric emergency department visits	0.4	(1.2)	0.3	(1.3)	0.3	(1.1)	0.6	(1.4)	0.3	(1.3)	0.5	(1.4)	0.4	(1.3)
Psychiatric outpatient visits	6.4	(12.2)	6.7	(15.5)	6.1	(13.8)	5.3	(10.0)	7.1	(16.5)	6.2	(12.5)	6.3	(13.4)
Injury-related outpatient visits	0.1	(0.4)	0.0	(0.3)	0.0	(0.3)	0.1	(0.7)	0.1	(0.4)	0.1	(0.5)	0.1	(0.5)
Non-psychiatric outpatient visits	2.9	(4.3)	1.4	(3.5)	2.0	(4.1)	3.2	(4.3)	2.0	(3.9)	2.7	(4.1)	2.5	(4.2)

Assessed in the 6-month period prior to the index drug fill.

a Indicates summary statistics for the index years of 2010 and 2011.

Aripiprazole was initiated in less than one in five individuals; haloperidol in only 6% of the cohort; and quetiapine in 26.5% (Table 1). Differences in cohort characteristics across the antipsychotic drug groups were apparent (Table 1); for instance, 43% of aripiprazole initiators had schizophrenia compared to 87% of haloperidol initiators, and 12% of haloperidol initiators had a psychiatric comorbidity compared to 22% of olanzapine initiators.

### Antipsychotic drug safety

Incident diabetes occurred in 9.3% and all-cause death in 5% of the cohort over the 3-year observation period (Table 2). While olanzapine was associated with the lowest unadjusted rate of diabetes (6.7%), aripiprazole was associated with the lowest unadjusted death rate (3.4%).

**Table 2.** Three-year outcomes among 38 762 publicly insured adults receiving antipsychotic drug monotherapy

	Individuals	Diabetes	Death
Aripiprazole	6686 (17.2)	679 (10.2)	225 (3.4)
Haloperidol	2328 (6.0)	217 (9.3)	103 (4.4)
Olanzapine	6301 (16.2)	421 (6.7)	313 (5.0)
Quetiapine	10 309 (26.5)	989 (9.6)	662 (6.4)
Risperidone	9897 (25.5)	941 (9.5)	470 (4.8)
Ziprasidone	3241 (8.3)	357 (11.0)	166 (5.1)
All	38 762 (100)	3604 (9.3)	1939 (5.0)

Number (percent) receiving each drug and having each outcome.

Adjusting for confounders, we estimate that if all patients were treated with haloperidol or olanzapine, their diabetes risk would be 1.9 (95% CI: 1.2–2.6) percentage points lower than if all patients were treated with aripiprazole (Table 3); the point estimate represents a 18.6 percentage point reduction compared with the unadjusted risk of diabetes among those treated with aripiprazole (10.2%, Table 2). There was a 0.5 (0.1–1.2) percentage point absolute reduction in diabetes risk favoring risperidone over aripiprazole, which represents a 4.9 percentage point relative risk reduction. We found no evidence of a relative safety benefit of aripiprazole over quetiapine or ziprasidone.

**Table 3.** Average absolute outcome differences in percentage points (95% confidence intervals) for five antipsychotic drugs compared with aripiprazole (comparator drug)

	Diabetes	Death
Haloperidol	−1.9 (−2.6 to −1.2)	1.1 (0.7–1.6)
Olanzapine	−1.9 (−2.6 to −1.2)	1.7 (1.2–2.1)
Quetiapine	−0.5 (−1.2 to 0.1)	2.2 (1.8–2.7)
Risperidone	−0.9 (−1.5 to −0.2)	1.6 (1.1–2.0)
Ziprasidone	0.3 (−0.5 to 1.0)	2.0 (1.5–2.5)

Positive values indicate an advantage for aripiprazole.

Aripiprazole appeared safer than all the other study drugs (haloperidol, olanzapine, quetiapine, risperidone, and ziprasidone) for the mortality endpoint. The estimated percentage point absolute risk increases for the five drugs ranged between 1.1 and 2.2 percentage points (Table 3), representing risk increases ranging between 32.4 and 64.7 percentage points relative to the unadjusted mortality risk among those treated with aripiprazole (3.4%, Table 2).

### Treatment effect heterogeneity

Findings across the diagnostic subgroups supported the main findings. Haloperidol and olanzapine reduced the risk of diabetes relative to aripiprazole for each diagnostic subgroup, with absolute risk reductions ranging between 1.8 percentage points (0.6–2.9) and 2.0 (1.1–2.9), for patients with bipolar I disorder and schizophrenia, respectively. Mortality effects were consistent with those in the overall cohort (see online Supplementary Table S1).

Findings across the racial/ethnic subgroups were generally consistent with the main findings (see online Supplementary Table S2).

### Sensitivity to unmeasured confounding

In analyses restricted to younger patients with no pre-period exposure to the index antipsychotic drug (*n* = 31 371), the diabetes advantages for haloperidol, olanzapine, and risperidone compared with aripiprazole found in the overall sample were not robust to the sensitivity analysis (Table 4). The main findings for mortality effects, with all study drugs having an increased risk compared with aripiprazole, were also not robust to the sensitivity analyses except for quetiapine, found to have an increased absolute mortality risk of 1.6 percentage points (0.4–2.7).

**Table 4.** Average absolute outcome differences in percentage points (95% confidence intervals) for five antipsychotic drugs compared with aripiprazole (comparator drug) for patients aged less than 45 years and no pre-period antipsychotic drug exposure

	Diabetes	Death
Haloperidol	−1.5 (−3.5 to 0.4)	0.9 (−0.3 to 2.2)
Olanzapine	−1.5 (−3.5 to 0.4)	1.3 (−0.2 to 2.7)
Quetiapine	−0.5 (−2.3 to 1.3)	1.6 (0.4–2.7)
Risperidone	−0.7 (−2.6 to 1.2)	1.2 (−0.1 to 2.6)
Ziprasidone	0.3 (−2.1 to 2.7)	1.5 (0.0–3.1)

Positive values indicate an advantage for aripiprazole.

## Discussion

The results of this study contribute to a large body of evidence on antipsychotic drug-related risks for non-elderly adults with SMI during real-world use. Previous research suggests that the FGA haloperidol, less frequently used than SGAs in the U.S. overall, has a favorable cardiometabolic profile relative to SGAs, while aripiprazole, a more widely used drug, has lower cardiometabolic risk relative to other SGAs (e.g. [Huhn et al., 2019; Pillinger et al., 2020]). However, their comparative risk has rarely been directly assessed. In this study, we observed a reduction in diabetes risk associated with initiating haloperidol, olanzapine, or risperidone treatment relative to initiating aripiprazole treatment. However, these diabetes advantages found in the overall sample, small for risperidone, did not hold in a sensitivity analysis that used a subgroup with a lower risk of confounding by indication. Mortality advantages of initiating aripiprazole relative to all study drugs were observed, but the only result that held in sensitivity analysis was the mortality advantage of initiating aripiprazole relative to quetiapine.

The similarity between our main estimates and estimates conditional on diagnosis is reassuring given that most studies have focused on schizophrenia populations; heterogeneity was plausible because each of these serious illnesses contributes to diabetes risk independently of antipsychotic drug exposures (Holt & Mitchell, 2015). While there are differences across racial/ethnic groups in how drugs are metabolized or tolerated (Horvitz-Lennon, Mattke, Predmore, & Howes, 2017), suggesting plausible mechanisms for effect heterogeneity by race/ethnicity, our analyses did not reveal effect modification by race/ethnicity.

This study is among the first to (a) leverage a doubly-robust machine-learning based method to assess the relative safety of antipsychotic drugs using observational data; (b) directly compare diabetes *and* mortality risk for aripiprazole and haloperidol, a drug regarded as having low cardiometabolic risk and, among individuals with dementia, a high mortality risk but with less evidence about their comparative risk in adults with SMI, as well as aripiprazole and several widely used SGAs, including olanzapine, a drug regarded as having high cardiometabolic risk, and (c) investigate whether SMI diagnosis and race/ethnicity modify the relative safety of these drugs.

### Limitations

Our study has some limitations. First, like all observational studies, unmeasured confounding may account for some of our results, including the absence of an aripiprazole advantage over the other SGAs. A potential source of unmeasured confounding is systematic drug assignment decisions based on prescribers' notions of the drugs' risks and unobserved patient risk factors. We lack the ability to identify prescribers (and leverage information on their prescribing patterns); however, we control for patient state of residence in the pre-period, which might absorb geographic variation in prescribing patterns. Moreover, we lack data on patients' medication adherence or lifestyle factors. While we control for pre-period differences in some risk factors for diabetes (obesity and other risk factors for cardiometabolic morbidity and utilization of drugs with cardiometabolic effects), we do not control for other risk factors such as patients' weight, smoking status, or family history. We note however that prior studies are similarly limited by observational designs. Additional limitations in prior studies include the specific comparisons made, e.g., SGAs *v.* any FGA (Citrome et al., 2007), antipsychotic drugs *v.* no use of antipsychotic drugs (Taipale et al., 2020), or antipsychotic monotherapy to antipsychotic polypharmacy (Katona et al., 2014), and their reliance on regression-based methods which are more vulnerable to confounding and model misspecification. Second, given the low risk of mortality (< 5%), we did not account for the competing risk of death in the diabetes analyses, nor did we take censoring into account. We do note that median follow-up was identical across drug groups, and mean follow-up was nearly identical. Third, because some drug subgroups defined by primary diagnosis or race/ethnicity were associated with small sample sizes, our method may have failed to detect real subgroup effects that exist within the population due to low statistical power. For example, the CIs of mortality differences with respect to race/ethnicity subgroups are wide, and in the setting of a low mortality rate and small mortality differences, they effectively rule out meaningful effects. Last, we did not adjust the CIs for multiplicity of estimation, so our findings should be replicated in similar cohorts.

## Conclusions

Relative to aripiprazole, quetiapine increased the 3-year mortality risk, a result that is consistent with some (Katona et al., 2014; Tang et al., 2022) but not all (Taipale et al., 2020; Tiihonen & Taipale, 2019) existing evidence. Although confirmatory research is needed, our findings suggest caution in the use of quetiapine in the care of non-elderly adults with SMI, even when used in low doses, as some evidence exists of the drug's risks at low dose (Berge, Abri, Andell, Movahed, & Ragazan, 2022). Also, we did not find that aripiprazole reduced diabetes risk compared to the other study SGAs including olanzapine, a finding that may be counter-intuitive for some observers but is in keeping with evidence produced by several previous studies (e.g. [Rajkumar et al., 2017; Smith et al., 2009; Stroup et al., 2009; Stroup et al., 2011; Vancampfort et al., 2016]). Ultimately, prescribing decisions should account for all potential SGA risks. Key among others, also associated with cardiovascular disease, are SGAs' risks for weight gain and dyslipidemia, for which aripiprazole and ziprasidone appear to have a clear advantage over other SGAs (Newcomer et al., 2008; Pillinger et al., 2020; Stroup et al., 2009, 2011; Weiden, Newcomer, Loebel, Yang, & Lebovitz, 2008; Wu et al., 2022).

## Supporting information

Poulos et al. supplementary material Poulos et al. supplementary material

## Note

1 The underlined words indicate confounder domains that for most are assessed through 1 variable (e.g. age domain is assessed through age variable) but some such require >1 variable (e.g. health status domain is assessed with 3 variables).

## Footnotes

† The note appears after the main text.

## Supplementary material

The supplementary material for this article can be found at https://doi.org/10.1017/S0033291723001502
