# Supplementary material for: Antipsychotics and the risk of diabetes and death among adults with serious mental illnesses
Source: Psychol Med. 2023 Sep 27;53(16):7677–84. doi: 10.1017/S0033291723001502 (PMC10758338; doi:10.1017/S0033291723001502)
Supplement: Poulos et al. supplementary material [file S0033291723001502sup001.docx]

**Supplementary Material**

**A. Analytic Plan**

**A1. Data Sources**

We used person-level Medicaid Analytic eXtract (MAX) administrative data extracted from each state’s Medicaid Statistical Information System (MSIS) and Medicare administrative data for calendar years 2008-2013 for California, Georgia, Iowa, Mississippi, Oklahoma, South Dakota, and West Virginia. The administrative data includes eligibility, demographic characteristics, diagnoses, and service and pharmacy utilization information. Continuous enrollment was defined as enrolled in Medicare Parts A (hospital services), B (outpatient services), and D (pharmacy).

**A2. Ascertainment of Diagnoses for Inclusion, Exclusions, and Variable Construction**

Diagnostic information was collected from billing claims indicating inpatient or outpatient encounters with medical professionals. If inpatient admissions were observed, we searched for diagnoses in claims in the Medpar (Medicare) and Inpatient (MAX/Medicaid) files. For outpatient encounters, we looked for diagnoses in outpatient and professional claims in the Outpatient and Carrier files (Medicare), and claims in the Other Therapy file (MAX/Medicaid). Because the MAX data include a maximum of two diagnoses per claim, we examined the first two Medicare diagnoses out of a maximum of 25 to balance the observable information among the payers.

SMI diagnoses required at least one primary inpatient discharge or two primary or secondary outpatient ICD-9 diagnostic codes in claims from two different days indicating *schizophrenia* (295.0−295.9), *bipolar I disorder* (296.0, 296.1, 296.4−296.7), or *severe MDD* (296.2x and 296.3x, with 5th digit indicating severe subtype with or without psychosis) during the 12-month period surrounding the index antipsychotic drug fill. Individuals lacking fifth digit codes met criteria if MDD codes were observed as primary discharge diagnosis in ≥ two occasions, or if ≥ one claim with electroshock therapy, trans-cranial magnetic stimulation, or suicide-related injury codes were observed during the 12-month period. Although SGAs are FDA-approved only for augmentation of antidepressant treatment, we did not require observation of a concurrent antidepressant because we might have missed antidepressant drugs prescribed prior to the six-months preceding the SGA fill and discontinued due to inefficacy or intolerance. Individuals qualifying for more than one of these diagnoses were assigned a single primary diagnosis based on phenomenological hierarchy (schizophrenia highest and severe MDD lowest), frequency, and recency of diagnosis (Charles et al., 2016).

We used ICD-9 diagnostic codes observed in the 6-month pre-period to create the health status variables and the conditions used for exclusions. We only required observation of one (1) claim, inpatient or outpatient, with a primary or secondary diagnosis.

We excluded individuals with Type 2 diabetes and other diabetes-related conditions such as secondary diabetes and diabetic polyneuropathy (Type 1 diabetes was not among them); dyslipidemia; hypertension; and cardiovascular disorders including coronary artery disease, heart failure, stroke & other cerebrovascular disorders, and other vascular disorders; and PCOS. See section B for ICD-9 codes for Conditions used for exclusions.

**A.3 Measures**

Outcomes.

Type 2 diabetes, ascertained with ICD-9 diagnosis codes for Type 2 diabetes (250.x0, 250.x2); because clinicians may have ascribed the new onset diabetes to ongoing antipsychotic treatment, we also included ICD-9 codes for secondary diabetes (249.x0, x1). We required (a) ≥ one primary inpatient discharge diagnosis or (b) ≥ two outpatient claims during a 12-month period with any of the qualifying codes as primary or secondary diagnosis, or one outpatient claim and one claim with a NDC for an oral antidiabetic drug. Insulin drugs were excluded as it was assumed that diabetes exclusively treated with insulin is Type 1 diabetes. The measure was assessed as of the 1st day following the index fill date until the end of the observation period.

All-cause death, defined based on variables denoting the person’s date of death available in the MAX and the Medicare datasets; the latest date of death was used in cases where these variables disagreed.

Subgroups

1. Primary Diagnosis (see A2): schizophrenia, bipolar I disorder, and severe MDD.
2. Race/ethnicity, assessed in the index month: Blacks, Latinxs, Whites, and subjects of other race/ethnicity a category that also included those with missing race/ethnicity information. We used the Research Triangle Institute (RTI) variable, one of the two race/ethnicity variables included in the Medicare data. The original variable captures self-reported race/ethnicity information collected by the Social Security Administration and transferred to the Centers for Medicare & Medicaid Services for inclusion in the Medicare program database. Because of incompleteness of this information, the Medicare program includes a second variable developed at the RTI that improves classification of Latinxs and Asians/Pacific Islanders through an imputation algorithm based on names from the US Census and geography (Eicheldinger & Bonito, 2008).

Confounders - assessed in the pre-period unless stated otherwise.

1. Age (continuous) and sex, assessed in the index month.
2. Health Status (binary yes/no variables): (a) other chronic medical conditions potentially associated with diabetes or with the potential to affect service utilization and thus likelihood of diagnosis (e.g., chronic infections); (b) risk factors for cardiometabolic morbidity (e.g., pre-diabetes); and (c) psychiatric comorbidity (other affective disorders, other psychotic disorders, anxiety disorders, OCD, and PTSD). See section B for details including the ICD-9 codes.
3. Service utilization: number of psychiatric, injury-related, and non-psychiatric inpatient days, number of ED visits, and number of outpatient visits.
4. Metabolic Testing: if ≥ one procedure codes for lipid or glucose laboratory tests were observed.
5. Exposure to drugs with cardiometabolic effects (binary yes/no variables using National Drug Codes): antidiabetic drugs, anti-hypertensive drugs, and other drugs with potential weight-related and cardiometabolic effects.
6. Exposure to the index antipsychotic drug.
7. Payer, assessed on the index date: dual eligible or Medicare based on coverage of the index fill.
8. Year of the index fill.

**A.4. Target Maximum Likelihood Estimation**

We utilized the sl3 package in R for constructing the treatment and outcome model ensembles as it supports multinomial classification algorithms and a multinomial loss function for the super learner (Coyle, 2021). A variety of flexible and nonparametric classification algorithms for the treatment and outcome model ensembles were selected: gradient boosting (Chen & Guestrin, 2016); random forests with varying forest sizes (Wright & Ziegler, 2017); L1-penalized lasso regression; L2-penalized ridge regression; and elastic net regressions weighting the L1 penalty at α ∈ {0⋅25, 0⋅50, 0⋅75} and the L2 penalty at 1 – α (Friedman et al., 2010).

**B. ICD-9 Codes**

Conditions used for exclusions

| 1. Dyslipidemia | 272.0, 272.1, 272.2; 272.4 |
| --- | --- |
| 2. Hypertension (essential) | 401.x |
| 3. Diabetes conditions |  |
| Type 2 Diabetes | 250.x0, 250.x2 |
| Secondary diabetes | 249.x0, x1 |
| Polyneuropathy in diabetes | 357.2 |
| Diabetic retinopathy | 362.0x |
| Diabetic cataract | 366.41 |
| Peripheral angiopathy, other | 443.81 |
| 4. Coronary Artery Disease |  |
| Acute myocardial infarction | 410.0x-410.9x |
| Other acute/subacute disease | 411.x [x=0,1]; 411.8x [x=1,9] |
| Angina pectoris | 413.0; 413.9 |
| Coronary atherosclerosis | 414.0x |
| Other coronary artery disease | 414.x [x=2-4] |
| Other chronic disease | 414.8, 414.9 |
| Old infarction & sequelae | 412, 429.79 |
| 5. Heart Failure |  |
| Heart failure | 428.xx |
| Heart failure with complications | 402.x1,404.x1, 404.x3 |
| 6. Stroke & Other cerebrovascular disorder |  |
| Intracerebral hemorrhage | 431 |
| Precerebral artery disease | 433.0x, 433.1x, 433.2x, 433.3x, 433.8x, 433.9x |
| Cerebral artery disease | 434.0x, 434.1x-434.9x |
| Transient ischemic accident | 435.x |
| Other cerebrovascular disorder | 436, 437.x [x=0,1,2,8], 438.xx |
| 7. Other Vascular |  |
| Hypertensive organ disease | 402.x0, 404.x0, 404.x2, 403.x0 |
| Cardiovascular disease, other | 429.2 |
| Atherosclerosis | 440.x [x=0,1], 440.2x, 440.3x, 440.4, 440.8, 440.9 |
| Peripheral vascular disease | 443.9 |
| Embolisms/thrombosis | 444.0x, 445.xx |
| 8. Polycystic Ovarian Syndrome | 256.4 |

Health Status

| **Other chronic medical conditions** |  |
| --- | --- |
| 1. Chronic Infections |  |
| Tuberculosis | 010-018 |
| HIV | 042 |
| Viral hepatitis | 070.2-070.9 |
| 2. Endocrine disorders |  |
| Goiter conditions | 240, 241 |
| Thyrotoxicosis plus/minus goiter | 242 |
| Hypothyroidism | 243, 244 |
| Thyroiditis | 245 |
| Other thyroid conditions | 246 |
| Juvenile Diabetes | 250.x1, 250.x3 |
| Other pancreatic conditions | 251; 577.1; 996.86, V42.83 |
| Other endocrine disorders | 226,227; 252-259 |
| 3. Malignancies | 140.xx–208.xx, 230.xx – 239.xx |
| 4. Lung disorders |  |
| COPD /Asthma | 490-496 |
| Pneumoconioses | 500-505 |
| Cystic fibrosis | 277.0 |
| Other lung disorders | 506.4 |
| 5. Gastro-Intestinal disorders |  |
| Ulcers | 531-534.9 |
| Liver conditions | 570-573 |
| 6. Renal disorders |  |
| Nephrotic syndrome | 581 |
| Chronic glomerulonephritis | 582 |
| Nephritis and nephropathy | 583 |
| Renal failure | 585, 586 |
| Renal sclerosis | 587 |
| Disorders from renal dysfunction | 588 |
| 7. Autoimmune, connective tissue & related disorders |  |
| Neuropathy (collagen disease) | 357.1 |
| Polyarteritis nodosa | 446.0 |
| Connective tissue disorders | 710 |
| Inflammatory polyarthropathies | 714 |
| Polymyalgia Rheumatica | 725 |
| Multiple sclerosis & related | 340, 341 |
| 8. Neurological disorders |  |
| Parkinson’s disease & related | 332.0, 332.1, 333 |
| Epilepsy | 345 |
| 9. Pregnancy-related |  |
| Diabetes complications | 648.0x |
| Abnormal glucose tolerance | 648.8x |

| **Risk factors for cardiometabolic morbidity** |  |
| --- | --- |
| Obesity & Overweight | 278.0x |
| Metabolic syndrome | 277.7 |
| Pre-Diabetes & related | 790.2x |

| **Psychiatric comorbidity** |  |
| --- | --- |
| 1. Affective Disorders |  |
| Neurotic depression | 300.4 |
| Cyclothymic disorder | 301.13 |
| Prolonged depressive reaction | 309.1 |
| Depressive disorder, other | 311 |
| 2. Other Psychoses |  |
| Paranoid states | 297 |
| Other nonorganic psychoses | 298 |
| 3. Anxiety disorders | 300.0x [x=0,1,2,9]; 300.2x [x=0,1,2,3,9]; 309.21 |
| 4. OCD | 300.3x |
| 5. PTSD | 309.81 |

**C. Supplementary Exhibits**

**Table S1. Average absolute outcome differences in percentage points [95% Confidence Intervals] for five antipsychotic drugs compared with aripiprazole (comparator drug), by primary diagnosis subgroup.** Positive values indicate an advantage for aripiprazole.

|  | **Diabetes** | | | **Death** | | |
| --- | --- | --- | --- | --- | --- | --- |
|  | **Schizophrenia** | **Severe MDD** | **Bipolar I disorder** | **Schizophrenia** | **Severe MDD** | **Bipolar I disorder** |
| Haloperidol | -1·9 [-2·9, -0·9] | -1·9 [-3·3, -0·5] | -1·8 [-2·9, -0·6] | 1·0 [0·4, 1·7] | 1·2 [0·3, 2·2] | 1·3 [0·3, 2·3] |
| Olanzapine | -2·0 [-2.9, -1·1] | -1·8 [-3·3, -0·3] | -1·8 [-3·2, -0·5] | 1·5 [0·8, 2·1] | 1·9 [0·9, 3·0] | 1·9 [0·9, 2·9] |
| Quetiapine | -0·5 [-1·5, 0·5] | -0·6 [-1.9, 0·7] | -0·6 [-1·7, 0·6] | 2·1 [1·4, 2·8] | 2·4 [1·4, 3·4] | 2·3 [1·5, 3·2] |
| Risperidone | -0·9 [-1·8, -0·1] | -0·8 [-2·2, 0·7] | -0·8 [-2·1, 0·5] | 1·4 [0·8, 2·0] | 1·8 [0·7, 2·9] | 1·8 [0·8, 2·8] |
| Ziprasidone | 0·2 [-0·9, 1·4] | 0·3 [-1.2, 1·9] | 0·2 [-1·1, 1·6] | 1·9 [1·2, 2·6] | 2·2 [1·1, 3·4] | 2·2 [1·1, 3·2] |

**Table S2. Average absolute outcome differences in percentage points [95% Confidence Intervals] for five antipsychotic drugs compared with aripiprazole (comparator drug), by race/ethnicity subgroup.** The ‘Other’ subgroup includes those with missing values for race/ethnicity. Positive values indicate an advantage for aripiprazole.

|  | **Diabetes** | | | | **Death** | | | |
| --- | --- | --- | --- | --- | --- | --- | --- | --- |
|  | **Black** | **Latinx** | **White** | **Other** | **Black** | **Latinx** | **White** | **Other** |
| Halop | -2·2 [-3·9, -0·4] | -2·2 [-4·6, 0·2] | 1·7 [-2·5, -1·0] | -2·0 [-5·1, 1·0] | 0·8 [-0·2, 1·8] | 0·8 [-0·7, 2·3] | 1·3 [0·7, 1·9] | 0·7 [-1·1, 2·5] |
| Olanz | -2·1 [-3·8, -0·4] | -2·1 [-4·4, 0·2] | -1·8 [-2·6, -1·0] | -2·1 [-5·1, 0·9] | 1·0 [0·0, 2·1] | 0·9 [-0·2, 2·0] | 2·0 [1·4, 2·6] | 0·9 [-0·9, 2·7] |
| Quetiap | -0·6 [-2·4, 1·1] | -0·3 [-2·7, 2·2] | -0·6 [-1·3, 0·2] | -0·6 [-3·5, 2·4] | 1·4 [0·4, 2·3] | 1·5 [0·3, 2·7] | 2·7 [2·1, 3·3] | 1·3 [-0·4, 2·9] |
| Risp | -1·1 [-2·7, 0·5] | -0·9 [-3·1, 1·3] | -0·8 [-1·6, 0·0] | -1·0 [-3·9, 1·9] | 1·0 [0·2, 1·9] | 1·0 [-0·1, 2·1] | 1·9 [1·3, 2·5] | 0·9 [-0·9, 2·7] |
| Zipr | 0·2 [-1·8, 2·2] | 0·4 [-2·2, 3·1] | 0·2 [-0·7, 1·1] | 0·2 [-2·9, 3·3] | 1·5 [0·4, 2·7] | 1·5 [0·1, 3·0] | 2·3 [1·6, 3·0] | 1·4 [-0·7, 3·5] |
| Halop= haloperidol, Olanz=olanzapine, Quetiap=quetiapine, Risp=risperidone, Zipr=ziprasidone | | | | | | | | |

| **Table S3**. STROBE CHECKLIST | Page  No. | Recommendation |
| --- | --- | --- |
| **Title and abstract** | 1-3 | (*a*) Indicate the study’s design with a commonly used term in the title or the abstract |
|  |  | (*b*) Provide in the abstract an informative and balanced summary of what was done and what was found |
| Introduction | | |
| Background/rationale | 4-6 | Explain the scientific background and rationale for the investigation being reported |
| Objectives | 6 | State specific objectives, including any prespecified hypotheses |
| Methods | | |
| Study design | 6-8 | Present key elements of study design early in the paper |
| Setting | 7 | Describe the setting, locations, and relevant dates, including periods of recruitment, exposure, follow-up, and data collection |
| Participants | 7-8 | (*a*) Give the eligibility criteria, and the sources and methods of selection of participants. Describe methods of follow-up |
|  |  | (*b*) For matched studies, give matching criteria and number of exposed and unexposed |
| Variables | 8-9;  Supplement A | Clearly define all outcomes, exposures, predictors, potential confounders, and effect modifiers. Give diagnostic criteria, if applicable |
| Data sources/ measurement | 6; Supplement A & B | For each variable of interest, give sources of data and details of methods of assessment (measurement). Describe comparability of assessment methods if there is more than one group |
| Bias | 9-10 | Describe any efforts to address potential sources of bias |
| Study size | 7-8 | Explain how the study size was arrived at |
| Quantitative variables | 9-10; Supplement A | Explain how quantitative variables were handled in the analyses. If applicable, describe which groupings were chosen and why |
| Statistical methods | 9-10; Supplement A | (*a*) Describe all statistical methods, including those used to control for confounding |
|  |  | (*b*) Describe any methods used to examine subgroups and interactions |
|  |  | (*c*) Explain how missing data were addressed |
|  |  | (*d*) If applicable, explain how loss to follow-up was addressed |
|  |  | (*e*) Describe any sensitivity analyses |
| Results | | |
| Participants | Supplement  Figure S1 | (a) Report numbers of individuals at each stage of study—eg numbers potentially eligible, examined for eligibility, confirmed eligible, included in the study, completing follow-up, and analysed |
|  |  | (b) Give reasons for non-participation at each stage |
|  |  | (c) Consider use of a flow diagram |
| Descriptive data | 10-11; Table 1 | (a) Give characteristics of study participants (eg demographic, clinical, social) and information on exposures and potential confounders |
|  |  | (b) Indicate number of participants with missing data for each variable of interest |
|  |  | (c) Summarise follow-up time (eg, average and total amount) |
| Outcome data | Table 2 | Report numbers of outcome events or summary measures over time |
| Main results | 11,12; Table 3 | (*a*) Give unadjusted estimates and, if applicable, confounder-adjusted estimates and their precision (eg, 95% confidence interval). Make clear which confounders were adjusted for and why they were included |
|  |  | (*b*) Report category boundaries when continuous variables were categorized |
|  |  | (*c*) If relevant, consider translating estimates of relative risk into absolute risk for a meaningful time period |
| Other analyses | 12; Table 4;  Supplement Tables S1, S2 | Report other analyses done—eg analyses of subgroups and interactions, and sensitivity analyses |
| Discussion | | |
| Key results | 13-14 | Summarise key results with reference to study objectives |
| Limitations | 14-15 | Discuss limitations of the study, taking into account sources of potential bias or imprecision. Discuss both direction and magnitude of any potential bias |
| Interpretation | 13-14 | Give a cautious overall interpretation of results considering objectives, limitations, multiplicity of analyses, results from similar studies, and other relevant evidence |
| Generalisability | 13-14 | Discuss the generalisability (external validity) of the study results |
| Other information | | |
| Funding | 1 | Give the source of funding and the role of the funders for the present study and, if applicable, for the original study on which the present article is based |

**Figure S1. Cohort selection flowchart diagram.**

**192,579**

Number of beneficiaries with SMI who had 6 months of continuous enrollment in the pre-period, were enrolled during the index month, and were enrolled either for 6 months post index month or until death

**159,839**

Number of beneficiaries with a qualifying monotherapy episode

**98,230**

Number of beneficiaries with no pre-period conditions used for exclusions

**64,120**

Number of beneficiaries in Medicare or Dual in the index month

**38,762**

Number of beneficiaries who were followed for up to three years (or died before the three-year follow-up)

**D. Supplementary References**

Charles, E. F., Lambert, C. G., & Kerner, B. (2016). Bipolar disorder and diabetes mellitus: Evidence for disease-modifying effects and treatment implications. *International Journal of Bipolar Disorders, 4*(1), 13. doi:10.1186/s40345-016-0054-4

Chen, T., & Guestrin, C. (2016). *Xgboost: A scalable tree boosting system*. Paper presented at the Proceedings of the 22nd ACM SIGKDD International Conference on Knowledge Discovery and Data Mining, San Francisco, California, USA. 785–794. doi: 10.1145/2939672.2939785.

Coyle, J., Hejazi, Nima, Malenica, Ivana, Sofrygin, Oleg, & Phillips, Rachael. (2021). Sl3: Modern super learning with pipelines (v1.4.4): Zenodo. doi: 10.5281/zenodo.5802288.

Eicheldinger, C., & Bonito, A. (2008). More accurate racial and ethnic codes for medicare administrative data. *Health Care Financing Review, 29*(3), 27-42.

Friedman, J., Tibshirani, R., & Hastie, T. (2010). Regularization paths for generalized linear models via coordinate descent. *Journal of Statistical Software, 33*(1), 1--22. doi:10.18637/jss.v033.i01

Wright, M. N., & Ziegler, A. (2017). Ranger: A fast implementation of random forests for high dimensional data in c++ and r. *Journal of Statistical Software, 77*(1), 1--17. doi:10.18637/jss.v077.i01
